# Bibliographical Notices

**Published:** 1838-01-17

**Authors:** 


### BIBLIOGRAPHICAL NOTICES.

*Mémoires de la Société Medicale d'observation, de Paris. Tome Premier.*

*Mémoire Analytique sur l'Orchite Blennorrhagique, par M. Marc d'Espine (de Génève) Docteur en Medicine, &c.*

*Memoirs of the Medical Society of Paris, Vol. 1. An Analytical Essay upon Blennorhagic Orchitis, by Dr. Marc D'Espine, of Geneva.*

Several enthusiastic votaries of medicine, disciples of M. Louis, whilst pursuing their studies in Paris, became convinced, that the uncertainty of their science, was, in a great degree, owing to the imperfect manner in which facts were observed and studied. Believing in the maxim of Hoffman, —ars medica tota in observationibus—they at the same time felt that this *observation* is, in itself an art, difficult in the extreme, and demands a long and arduous apprenticeship. This led them to conceive the idea of an association, having for its objects, to overcome these obstacles by meeting them, to render observation useful, by making it exact, and to point out the mode of generalizing with safety from particular facts.

Such was the origin of the Medical Society of Observation of Paris, which was organized, by the appointments of M. Louis, as perpetual President, and M. M. Andral and Chomel, as honorary Presidents. Among its members are numbered several of our own countrymen, already distinguished for their devotion to medicine.

This is no holiday association, but one which exacts a practical illustration of the precepts which it inculcates, from all those who participate in its honors. It permits, with a solitary exception,* no honorary members, and allows no one to assume the title of *Fellow*, who has not participated in its active duties, for at least six months. The resident members assemble once a week, at which time one or more observations are contributed, which undergo a rigorous scrutiny, not only upon their prominent points, the omissions, or the propositions announced, but more especially upon the means employed to verify the assertions.

The volume before us, which is the first number of their transactions, contains several highly interesting, and very elaborate essays, prepared with all the nicety and vigilance, so characteristic of the school, which claims as its head the illustrious Louis. Translations of three of these, have, already, been presented to the American public, by members of the Society. We trust before long to see the complete work, in the hands of every practitioner and student.

We have selected, for the purposes of brief analysis, one of the as yet untranslated memoirs, on a subject which has not hitherto ever been philosophically investigated. It is contributed by Dr. Marc D'Espine, and examines very thoroughly the history, &c., of Orchitis.

The unmeaning and unpathological cognomen of Hernia Humoralis, has been conferred upon those swellings of the testicle, supervening upon an attack of gonorrhœa, although not invariably arising from that cause; the same condition being sometimes induced by cold or external violence, operating on the healthy gland. Since the adoption of a more correct nomenclature, the term Orchitis—from ὀρχις, testicle has been substituted. M. D'Espine, from its being, in his opinion, almost always dependent upon urethritis, no matter how produced,—whether the effect of a specific virus, or any other irritant—prefers the name of Blennorhagic Orchitis; it conveying, according to his notion, a more correct idea of the disease.

An analysis of thirty cases of swelled testicle, observed during the year 1832, at the Venereal Hospital of Paris, form the data of this essay. These cases were not especially selected, but were taken indiscriminately. They include a solitary instance of Orchitis not blennorhagic, but which was attended with ulcerations upon the prepuce. This M. D'Espine thinks, goes far towards establishing the greater frequency of the blennorhagic variety of the disease. Great care was taken in the interrogations of the subjects of these cases, and any possibility of the desired answers being anticipated, was particularly avoided. This verbal examination was sometimes prolonged to one hour and a half. The memoir is divided into four parts. The first treats of the symptoms; the second of the causes; the third of the treatment; and the fourth contains some remarks upon Orchites not blennorhagic, with a *resumé*. We shall endeavor to follow our author through the several divisions of his subject, noting carefully the most important points.

This affection may supervene at any period of urethritis. It may appear simultaneously with it, or commence sometime during the acute state of the discharge, or only towards the termination of the disease, when it may have existed for months, or even years. It rarely occurs however before the *eighth* day of gonorrhoea. The principal circumstances which conduce to an attack, are, muscular motion, the employment of astringent medications, with a view of suddenly arresting the flux, and moral emotions; although it occasionally happens without the intervention of any of these causes.

The manifestation of Orchitis is sometimes accompanied with an entire suppression of the gonorrhoeal discharge, which re-appears at the end of three or four days, even before the acute symptoms of urethritis have been checked; sometimes the suppression eventuates as a complete cure, no restoration of the discharge taking place. Generally however the suppression is preceded by a gradual diminution, which begins usually about the commencement of the Orchitis, and sometimes at a more remote period. Occasionally it is a simple decrease in the quantity, and continues so throughout the whole course of the disease. It is sometimes, though rarely, intermittent. Very often no modification whatever occurs.

Whatever may be the cause, its invasion is, for the most part, characterised by a painful and disagreeable tension about the scrotum and groins. Tumefaction rarely precedes the pain. Very soon though the testicle and chord acquire a considerable volume, and at the same time the pain increases. At this period the general system sympathises, and more or less intense febile movement accompanies the local lesion. A gradual increase of all these symptoms takes place, for several days; but by the seventh or eighth, they begin to decline. In this disappearance they usually observe the following order. 1st. The constitutional affection; 2nd. the pain; and lastly, the tumefaction. If you examine carefully, at the onset of the disease, the scrotum, you are unable to distinguish the epidydimis, amidst the general swelling. Before long, the tumour becomes indurated in the situation of the epidydimis, and soon the testis itself, becomes involved. Two distinct masses are now appreciable as hard as stone; the one posterior and inferior, being the epidydimis; the other superior and anterior, being the body of the testis. At length the testicle returns to its normal size and consistence. The induration of the epidydimis is sometimes completely resolved in a few days; but very often it remains hard for a long time; months or even years.

* Marshall Hall, M. D., of London.

Orchitis does not invariably pass through all these stages, and resolution often occurs before even the epidydimis becomes distinct.

Its course too may be interrupted by one or more relapses; and this accident may happen at any period of the disease. Generally this is announced by a return of pain, which is quite or nearly as severe, as at first; the swelling too reappears; the epidydimis again becomes distinct; and, if the pain be considerable, there is a return of the febrile symptoms. The causes of a relapse are commonly the same as those which tend to produce an attack; sometimes though they are inexplicable.

The *Invasion* was observed in 22 cases; 13, had pain; 3, swelling; 6, both pain and swelling. The mean age of those in whom the disease commenced by pain, was 26 years; ditto by tumefaction, 25 years; ditto both united, 23 years. In 20 cases, of 9 left orchites, 6 began with pain alone, and 3 with tumefaction with or without pain. Of 11 right orchites, 6 had pain alone at the commencement, and 5 swelling with or without pain.

*Pain.*—In 13 cases where pain was the first symptom, its seat was not invariable. In 9 it was in the groin of the affected side; and in 3 others, about the testicle; and 2 of these localized their pain at the inferior and posterior portion of the gland. The last experienced a dragging sensation of great weight in the testis rather than acute pain. As pain is, in general, the first symptom to manifest itself, so is it the first to disappear. It is usually only severe during the first few days, and then it is so acute, that the patient scarcely knows what position to assume. Pain or pressure remains after the subsidence of spontaneous pain; yet it is very difficult to limit the precise period when one ceases, and the other alone continues. The cessation of pain should be estimated from the period, when moderate pressure is not attended by more sensation than in the healthy condition of the organ.—The duration of the pain, thus limited, gives a mean of about 21 days; that is 12 to 13 days for double orchitis; 21 to 22 days for the right, and 24 to 25 days for the left. Thus the right orchites are at once those where pain is most commonly the earliest symptom, and where it continues longest. The reverse obtains in double orchites. M. D'Espine thinks that the swelling does not follow the same law. Not only are right orchites, those in which pain most generally ushers in the symptoms, and remains the longest time, but it is in them that it is of the greatest intensity. The double orchites, on the contrary, are not only less painful but are so for a shorter period.

The strength of the constitution exercises a marked influence upon the degree of pain. It would appear from an observation of 27 cases, that it is in inverse ratio to the forces; subjects who are meager and lean suffering most.

With regard to the influence which the duration of the discharge exercises upon the violence of this symptom, it was found to be greater when the former was of long standing.

*Tumefaction.*—Our author next proceeds to examine the seat, progress, development, the circumstances which influence the march, and duration of the swelling; also its consistence.

The tumefaction occupies the scrotum, testicle, epidydimis and cord. That of the scrotum exists from the onset. M. D'Espine divides the progress of the swelling into three degress. The *first degree* is where the tumor is equally hard every where, and forms a hymogeneous whole, no division being apparent between the epidydimis and the testis itself. This having lasted for several days, another modification ensues; the posterior border of the tumor augments in hardness, whilst anteriorly it becomes elastic (renitente.) This constitutes the second stage. The consistency of the epidydimis is now increased near its free border, but you still are unable to define the exact line of its separation from the testicle. More slowly the induration advances towards the centre of the tumor, and bounds itself exactly by an oblique line, the direction of which is from above downwards, and from behind forwards. This divides the glandular tumor into two portions; the anterior and superior portion being the body of the testis, which, by this time is nearly reduced to its normal consistence and volume. This is the third stage. The epidydimis is now diminished in bulk, its hardness, though, being preserved, and its surface being uneven and knobbed. The line of separation becomes now more and more defined, and at length the epidydimal tumor presents itself as a hard knot, entirely isolated from the testicle. Such are the appearances of the 4th stage. You sometimes meet with two of these bosses, placed one above the other. When there are a number, their location is intermediate to these two. For the most part one only exists, and it is situated posteriorly and at the base of the tumor. Nothing now remains but complete resolution, which is often delayed for a long time.

It must be borne in mind that the tumefaction does not in its progress, observe an invariable routine. With some, resolution is accomplished before the epidydimis becomes distinct. Sometimes, after having reached the second stage, it is discussed, without further progression; or on reaching the second, it passes by the third, and recommences at the end of the fourth stage, or if it has existed it was so slight that it was unrecognized. You also see, though rarely, the tumor present during the second or third stages, a character directly opposite to that just described. For instance; after the disappearance of the acute symptoms, two well marked divisions are evident, distinguished by the difference of their consistence; but in this case it is the superior and anterior portion—the body of the testis—which is hard and bossed, the epidydimis being soft and doughy. This happened three times in twenty-nine cases; and in two of these the tumor had returned into the ordinary state; the testicle, which was hard becomes now soft, and the epidydimis, which was doughy and elastic, becomes indurated.

The preceding details are rendered certain by the subjoined analysis of the facts.

*State of the epidydimis during the four first days.*—On account of the patients not resorting to the hospitals until after the disease has existed for several days, the number of observations at this period are limited. *Five* only are recorded; in 3 of these the epidydimis was not at all distinct; in the remaining two the second stage had commenced; the tumor being harder posteriorly than elsewhere.

*From the 4th to the 10th day.*—In 14 cases—those cases in which relapses occurred previous to this period, and those exhibiting evidences of former attacks, being omitted—6 were still in the 1st stage; in the remaining 8 the epidydimis was indurated, or, in other words, the 2nd stage had commenced.

*From the 10th to the 20th day.*—10 cases; not one in the 1st stage; 6 in the 2nd; 2 in the 3d; 1 in the 4th; and 1 completely cured.

*From the 20th to the 40th day.*—First stage 0; 2nd, 3; passing from 2nd to 3rd, 4; 3rd, 1; 4th, 2; epidydimis resolved, 6.

*After the 40th day*, the majority of the patients either left the hospital sick or were cured before the 45th day; 2 only went out on the 35th and the 56th day, and in both the epidydimis was still indurated.

From the foregoing facts the following laws are deduced. *In blennorhagic orchitis the epidydimis is invariably implicated. It may present four different stages of alteration, during the course of the disease. If its progress is interrupted by no relapse, and if each stage runs its full course, they always succeed each other in the same order. Resolution may at any period supervene; but it is the more slow, as the disorder becomes more advanced.*

In the six cases of double orchitis,* which came under observation, resolution of the epidydimis occurred, in the first stage; in the two cases of originally double orchitis, and in that one of the remaining cases which most resembled simple orchitis; while in the cases of an intermediate character, the epidydimis underwent the transformations of one or more stages, before resolution took place. This law, which seems to bring together the two extremities of the ladder so far as relates to the morbid progress of the epidydimis, would be of much interest, if confirmed by further observations.

*State of testicle from the first to the fourth day.* Five cases were noted; the gland was enlarged and indurated in four; in the fifth, it was already soft and of the natural size.

*From the fourth to the tenth day.*—Fourteen cases; in three the testis had returned to its normal state; in two others it was about twice the healthy dimensions; in one of these two, it was still hard; in the other it was elastic, (renitent.) In the remaining nine it was still of very considerable volume, varying from the size of an egg to that of the fist. In these nine cases the testis in two was elastic; in the seven others it had all the usual hardness.

*From the tenth to the twentieth day.* In ten subjects the testicle was resolved in two; in one case only was it still of double size; in seven others it was not more than five-sixths larger than natural. With respect to the consistence, in eight cases uncured, it was normal; two were elastic.

*From the twentieth to the fortieth day.*—Sixteen cases; testis natural in ten; in three consistence good, but they are still enlarged; in three others it was rather firmer, and more developed than natural.

We thus find that the resolution of the testis is more rapid than that of the epidydimis, since we have an example of resolution in the first period. We also see that the chances of complete resolution in the first forty days, are much greater for the testis than for the epidydimis; since at the fourth stage, five-eighths of the testis are resolved, whilst at the same period we have the cured epidydimis amounting to three-eighths only.

Our author thinks himself justified from all the facts, in establishing the following laws. *The resolution of the epidydimis is either always subsequent to that of the testis, or occurs pari passu with it, but never precedes it; so that a complete cure can never occur until the body of the testis has returned entirely to its natural condition. After the pain, the induration of the testicle, is the next symptom to disappear; then the tumefaction. Precisely the contrary obtains in the case of the epidydimis.*

*State of the cord from the first to the fourth day.*—In five cases observed at this period, two only furnish any information of the state of the cord. In these it was hard and tumefied.

*From the fourth to the tenth day.*—eight cases noted; in one the cord is healthy; in four it is very hard and much swollen; in the remaining three the tumefaction is slight.

*From the tenth to the twentieth day.*—Ten cases; three natural; two yet hard and tumefied; in five others it offers an intermediate condition.

*From the twentieth to the fortieth day.*—twelve cases; cord sound in four; in one only is there much hardness and swelling; in the remaining seven it is very slightly beyond either the natural size or consistence.

In reviewing these results we find no instance of resolution of the cord during the first stage; in the second, we have one in eight cases; in the third, three in ten; in the fourth, four in twelve. Thus then, *the resolution of the cord is intermediate to that of the testicle and the epidydimis.*

*The scrotum.*—The external position of this envelope, subjects it as much to the influence of the various topical applications employed, as to that of the disease, thus rendering it difficult to distinguish the effects of each. But M. D'Espine is disposed to consider the vermil tint, heat, and thickening, with the peculiar disposition to adhesion to the subjacent tissues, causing the disappearance of the rugæ, as dependent on the pathological condition, whilst the violet color, like the skin of an onion, the papular state acccompanied with intense itching, the furfuraceous desquamation, and the œdema without any increase of temperature, as caused by the topics.

Our author in concluding this portion of his subject, gives the subjoined table, exhibiting the proportion of cures during the different stages, in the four organs enumerated.

Cures from the first to the fourth day.

Testicle, 1-5; Cord, 0; Epydidymis, 0; Scrotum, 1-4.

* The term *double orchitis* is not only applied to cases, in which both testicles are simultaneously affected, but also to those, in which one is attacked before the other is cured.

From the fourth to the tenth day.

Testicle, 3-4; Cord, 1-8; Epidydimis, 0; Scrotum, 1-6.

From the tenth to the twentieth day.

Testicle, 2-10; Cord, 3-10; Epidydimis, 1-10; Scrotum, 1-2.

From the twentieth to the fortieth day.

Testicle, 10-16; Cord, 4-12; Epidydimis, 6-16; Scrotum, 11-15.

*General and sympathetic symptoms.*—These only attend the acute stage of the disease; when amelioration of the local lesion occurs, they vanish: the least relapse though, causes their reappearance. In twenty-five persons five experienced no fever whatever; two others had none at the commencement, but the relapses were attended by general symptoms; in the five *sine febre*, two had relapses, which were also apyrexic. So that in one fifth of the cases, orchitis was not complicated with fever, at any stage, and that in a little more than one-third the acute stage passed without fever, but appeared during relapses; so tha the absence of the constitutional affection at the onset, does not warrant us in concluding that it will not subsequently supervene; although we have seen one or two relapses without pyrexia, succeeding to an attack equally unattended by general symptoms.

In twenty-seven cases on the contrary, the acute stage was accompanied by general symptoms of variable intensity and duration. They continued in one case, even to the thirteenth day; in another they disappeared after the first day, although scarcely less intense. Between these two extremes was the duration of the remaining fifteen.*

The duration and intensity of the febrile movement, appears to be dependent on those of the pain; of four cases where there was little or no pain, there was a total absence of fever, or very nearly so. The contrary obtained where there was much intensity of pain.

Fever continues longest in the left orchitis, the right comes next, it disappearing the soonest in the double; the mean for each being five, three, and two and a half days. In robust subjects it lasted two and a half days, and in the feeble ones five days, as a mean number.

In eight cases of relapses observed, fever was absent in two. In two others the fever was very severe, and what is curious, they were unaccompanied by fever originally. In the remaining four, the pyrexia was moderate. The proportion of cases uncomplicated with fever is the same for the relapses as the original attacks.

M. D'Espine includes in the sympathetic phenomena, all those symptoms which are beyond the seat of the local affection, and which do not in themselves constitute any of the elements of fever. Three individuals complained of pain in the lumbar region; two at the commencement of the attack; and one during a relapse. Four experienced intense pains shooting to either the thigh, groin, or anterior iliac spine of the side of the affected testicle. Our author supposes these pains to be neuralgic—analagous to those succeeding to the operation for cataract—the ramifications of the lombo-abdominal plexus being involved. Yet from their rare occurrence he is inclined to suspect the existence of some peculiar exciting cause.

*Duration and termination.*—The fever yields first, its mean duration being a little more than five days. After this the pain terminates; its mean duration being from twenty to twenty-one days. The mean duration of the tumefaction and induration exceeds considerably these numbers. More than half of the patients leave the hospital, before they are completely well. Of fifteen cases in which he was enabled to observe the termination of these symptoms—and consequently of the whole affection—it varied from eighteen days for the *minimum*, to fifty-three days for the *maximum*. This gives a mean of twenty-three days for orchitis, cured under his own eye.

The mean duration of cases uncomplicated with relapses, and those, in which one or more relapses occur, is thirty-one days for the first, and thirty-four days for the second. If the relapses appear to retard somewhat the cure, their influence is much less *than a priori* you would be induced to believe.*

Of the fourteen cases who quitted the hospital, before a complete cure was effected, the majority left between the twentieth and the fortieth day. two remained until the sixty-seventh and eighty-seventh days, and in both there was induration of the epidydimis. This condition, as previously remarked, persists a long while. In the wards of the *Hospital de Veneriennes*, there is a man, who has on the posterior portion of one of his testis, an insolated, indolent tumour, of stony hardness, the vestige of an orchitis, happening two years previous.

M. D'Espine asks, whether this induration of the epidydimis can be considered as one of the terminations of orchitis? He decides not, since it is capable of resolution.

He considers the terminations by chronic tumour, (sarcocele) suppuration and gangrene to be very rare. In the twenty nine cases analysed, not one instance of these terminations occurred, although several of them had been several times previously afflicted. The experience of M. Cullorier, confirms M. D'Espine, in an experience of several hundred cases, he never saw more than two or three terminate by suppuration.

M. D'Espine concludes Part first of his memoir by a careful analysis and comparison of the opinions of the different authors upon the symptoms, progress, and termination of blennorhagic orchitis.

*To be Continued.*

* In six they disappeared on the third day; one on the fourth; one on the fifth; two on the sixth; two on the seventh; two on the eighth; and one on the ninth.

* The mean duration of left, right, and double orchitis, is twenty-nine days for the first and second; and thirty-eight for the third. Thus the double orchitis are at once less painful, are accompanied more rarely with fever, and are the longest to cure.

*A Practical Treatise on the Diseases of the Skin, arranged with a view to the Constitutional Causes and Local Characters, &c. By Samuel Plumbe. Fourth Ed. London*, 1837. *Philadelphia, Select Medical Library, Haswell, Barrington & Haswell*, 1837.

This excellent treatise upon an order of diseases, the pathology of which is in general as obscure as the treatment is empirical, has just been re-published in the Select Med. Library, edited by Dr. J. Bell of this city. We hail with pleasure the appearance of any new work calculated to elucidate the intricate and ill-understood subject of skin-diseases. The late Dr. Mackintosh, in his Practice of Physic, recommends it as the "best pathological and practical treatise on this class of diseases, which is to be found in any language."

Several reprints of great value have already appeared in the Library—among others we may mention, Pritchard on Insanity, Curling on Tetanus, Latham's Clinical Lectures, &c. The No. for the present month commences Collins' Treatise on Midwifery, a work rich in statistical details.

---

*A Clinical Lecture on the Primary Treatment of Injuries, delivered at the N. York Hospital, Nov 22d*, 1837: *By Alexander Stevens, M. D. &c., pp.* 34. *N. York, Adlard & Saunders*, 1837.

The mass of practical matter embodied in clinical lectures, where the disease under consideration is illustrated in all its various phases by cases, is usually lost, or is available only to the favored few who compose the auditory, or the still smaller number who recollect it. Entertaining a deep sense of the utility of these lessons of experience, and of the importance of preserving and offering them to the medical public at large, we were induced to commence our present undertaking. The publication of the above lecture by Professor Stevens, we notice with peculiar satisfaction, more particularly as it is an earnest of future contributions from the same valuable source.

The subject of this lecture is one of infinite importance, but one which has unaccountably been almost overlooked by surgical writers. It treats of daily occurrences, from want of proper attention to which, the surgeon will find himself repeatedly foiled in his best directed efforts. "A simple and practical account of the nature and treatment of the primary symptoms of severe injuries," has been long imperatively demanded, and this little work most ably fulfils the indication. Dr. Stevens gives first a general outline of the symptoms, and then briefly considers the etiology and prognosis. His observations on the treatment are of intrinsic value; they are simple, judicious and practical. He very properly animadverts upon the uncalled for and ill-timed interference too frequently made by practitioners, with symptoms and diseases, which are self-limited, and if unmolested would probably terminate favorably. How often, indeed, is the physician's whole duty necessarily restricted to a simple watching of the progress of the malady, prepared though he be to notice and meet any untoward symptoms that may arise. He is in the light of an Esquire in the conflict between nature and death; as equerry to the first, his duty is to remove at once all opposing obstacles, and thus facilitate a happy issue. Or, as Dr. Stevens tells us, "the physician does not propel the boat, but keeps it from the rocks and quicksands in its course."

We heartily concur with our author in his condemnation of the routine and empirical practice of invariable bleeding on the receipt of a severe injury, especially where the brain is implicated, previous to reaction. Such a course, we are convinced, hastens the very end which it is desirable to avoid; accelerating, instead of retarding the accession of inflammation.

We regret that our space does not permit us to analyse more fully this excellent little work; from what has already been said though, we are confident our readers will not be satisfied until they possess and read it themselves. We cannot conclude without adding one more quotation pregnant with excellent advice.

"I trust, gentlemen, you will distinguish between the coolness and self-possession which every surgeon should possess, and that heartless manifestation of indifference, which allows him to think of the figure he himself is making, at a time when all the energies of his mind should be exclusively directed to the welfare of his patient."

